# The Effect of Cigarettes and E-Cigarettes on Epithelial-Derived Extracellular Vesicles: A Systematic Review

**DOI:** 10.3390/ijms27062787

**Published:** 2026-03-19

**Authors:** Rute Santos, William Browne, Amanda Tatler, Victoria James, Lucy C. Fairclough

**Affiliations:** 1 School of Life Sciences, University of Nottingham, Nottingham NG7 2RD, UK; mbxrs11@nottingham.ac.uk (R.S.); msxwb2@nottingham.ac.uk (W.B.); 2 School of Medicine, University of Nottingham, Nottingham NG7 2RD, UK; amanda.tatler@nottingham.ac.uk; 3 School of Veterinary Medicine and Science, Biodiscovery Institute, University of Nottingham, Nottingham NG7 2RD, UK; victoria.james@nottingham.ac.uk

**Keywords:** cigarette, e-cigarette, extracellular vesicles, lung inflammation, epithelial cells

## Abstract

Extracellular vesicles (EVs) are lipid-enclosed particles secreted from a wide variety of cells, with the ability to transfer biologically active content from parent to recipient cells. Lung epithelial-derived EVs (LE-EVs) play an important role in the progression of pulmonary disease, but there is limited evidence regarding the impact of cigarette smoke (CS) and electronic cigarette aerosol (ECA) on epithelial-derived EVs. The aim of this systematic review was to evaluate the current published literature on the impact of cigarette smoke and electronic cigarette aerosol on LE-EVs. Original research studies and clinical data were included, but research involving microparticles or non-epithelial-derived EVs was excluded. A total of 29 articles were identified from three databases (EMBASE, Web of Science and PubMed), of which nine demonstrated that CS exposure leads to molecular changes in epithelial-derived EVs, whereas 21 reported that CS-induced LE-EVs can deliver their cargo to neighbouring cells. The results highlighted that LE-EVs secreted in response to cigarette or e-cigarette exposure presented altered EV cargo, associated with increased cellular damage, inflammation and disease development. The current literature suggests that conventional and electronic cigarettes can influence the secretion of EVs from lung epithelial cells, with these EVs potentially playing a role in the development of lung inflammation. Nonetheless, there is limited research studying the impact of ECA on LE-EVS. Further research examining the impact of electronic cigarettes on lung epithelial-derived EVs, using robust human in vitro models coupled with clinical studies, is required.

## 1. Introduction

The use of tobacco products poses one of the biggest global threats to human health, causing over 7 million deaths annually, of which over 1.5 million are credited to second-hand smoke exposure [1,2]. According to recent World Health Organization (WHO) estimates, 1.3 billion people worldwide use tobacco products, including conventional cigarettes and heated tobacco products (HTPs) [1]. Electronic nicotine delivery systems (ENDSs) were developed as tobacco-burning alternatives, but they typically contain nicotine, the principal active constituent of tobacco. Conventional cigarettes contain approximately 600 compounds, and when burnt, they reach temperatures as high as 900 °C, releasing over 7000 chemicals, including tar, nicotine, and nitrogen (3–6) [3,4,5,6]. HTPs are tobacco products that heat tobacco sticks at lower temperatures, usually between 250 and 350 °C, but can go as high as 550 °C [6,7]. Unlike conventional cigarettes or HTPS, ENDs (such as electronic cigarettes or e-cigarettes) do not contain tobacco leaves; instead, they use an e-liquid solution, consisting of a mixture of propylene glycol (PG) and vegetable glycerine (VG) that can contain nicotine (from tobacco or synthetic nicotine), as well as synthetic flavours [8]. E-cigarettes reach lower temperatures than HTPs, usually 200–250 °C, and generate an aerosol that releases over 30 chemicals [7].

It is well documented that conventional smoking and exposure to second-hand smoke significantly increase the incidence and risk of mortality of pulmonary diseases, such as chronic obstructive pulmonary disease (COPD), asthma, lower respiratory infections, and cancer (trachea and bronchus) [4,9,10,11,12,13,14]. Even though e-cigarettes have been in use since 2003 [15], there is limited evidence regarding the long-term effects of HTPs and ENDs on the development and progression of pulmonary diseases such as COPD and lung cancer. Nonetheless, recent evidence suggests that the use of e-cigarettes increases the incidence of respiratory symptoms (coughing, coughing up mucus, feeling out of breath, wheezing, and easily getting a cold) in young adults [16,17], as well as increasing the risk of cardiovascular diseases by reducing myocardial and vascular function [18,19].

The airway epithelium acts as the first line of defence against inhaled particles from the external environment, such as cigarette smoke (CS) and e-cigarette aerosols (ECAs) [20]

The lung epithelium consists of tightly packed epithelial cells held together by tight junctions (TJs), adherens junctions (AJs) and desmosomes. These cell junctions confer the epithelium’s selective permeability and promote cell-to-cell and cell-to-extracellular matrix adhesion communication [21,22]. Additionally, the lung epithelium is responsible for maintaining lung homeostasis by promoting tolerance and limiting inappropriate immune responses [23]. This epithelial-immune cell crosstalk is essential in the development of lung inflammation and the repair of lung injury [23,24]. Loss of epithelial barrier integrity due to cleavage of cell junctions results in barrier dysfunction, characterised by hyperproliferative epithelial cells, aberrant cell-cell interactions, and uncontrolled leakage of inhaled compounds across the airways, ultimately leading to abnormal immune responses [25,26].

Extracellular vehicles (EVs) are lipid bilayer membrane-contained particles that are unable to self-replicate [27]. EVs range from 30 nm to 10 μm in size and can be subdivided into small EVs (<200 nm in diameter) or large EVs (>200 nm in diameter) [27,28]. According to their biological content, biogenesis and cellular origin, EVs can be further classified as exosomes, microvesicles, apoptotic bodies and autophagic EVs [27,28,29,30,31]. EVs are secreted from a wide variety of cells, including epithelial cells, mesenchymal stem cells (MSCs), immune cells (dendritic cells, T cells, B cells, macrophages), and have been found in various bodily fluids (blood, bronchoalveolar lavage fluid, sputum, and urine). Once secreted, EVs retain properties of the parent cell, thus reflecting its physiological condition [32,33]. EVs can transfer their biologically active content (proteins, lipids, nucleic acids and sugars) from parent cells to recipient cells (local or distal), ultimately altering the target cell fate. This capacity to transport and deliver cargo to target cells grants EVs the ability to influence physiological processes, as well as to modulate downstream immune responses, thus making them potent mediators in intercellular communication [28,29,30,31,34]. In the lungs, EVs are involved in homeostasis maintenance and have been shown to influence the progression of pulmonary diseases, such as COPD and asthma [35,36].

There is emerging evidence suggesting that lung epithelial-derived EVs play an important role in the progression of pulmonary disease [37]. Furthermore, epithelial-derived EVs have been identified as potential therapeutic targets against respiratory diseases [38,39]. Nonetheless, there is limited evidence regarding the impact of CS and ECA on epithelial-derived EVs. Understanding the impact of CS and ECA on epithelial-derived EVs, as well as the subsequent effects of CS- or ECA-induced epithelial-derived EVs on early immune responses, could help elucidate the initial mechanisms that underpin the early events in lung inflammation. In turn, this could provide the potential to better inform new approaches to prevent the development of smoking/vaping-related respiratory diseases, as well as provide insight into future therapeutic strategies. This systematic review, therefore, aims to investigate the current evidence of the impact of cigarettes and e-cigarettes on epithelial-derived EVs, both in EV cargo and the subsequent downstream effects.

## 2. Methods

### 2.1. Search Strategy

This review was conducted in compliance with the Preferred Reporting Items for Systematic Reviews and Meta-Analyses (PRISMA) guidelines. To identify relevant articles, three databases, EMBASE, PubMed and Web of Science, were searched and filtered to include only original research articles, as well as articles published in English.

The key search terms were as follows: “Epithelial,” “Epithelium,” “Epithelial cells” and “Extracellular Vesicles,” “EVs,” “Microvesicle,” “Exosome,” “Ectosome,” “Shedding Vesicle,” “Microparticle” and “Lung” and “Cigarette smoke”, “Smoking”, “Tobacco smoke”, “Tobacco”, “E-cigarette” “E-cigarette vapour”, “E-cig”, “E-cigarette vapor”, and “Vaping”. Terms were searched using the advanced search settings for each database, as per Supplementary Document.

**Figure 1:**
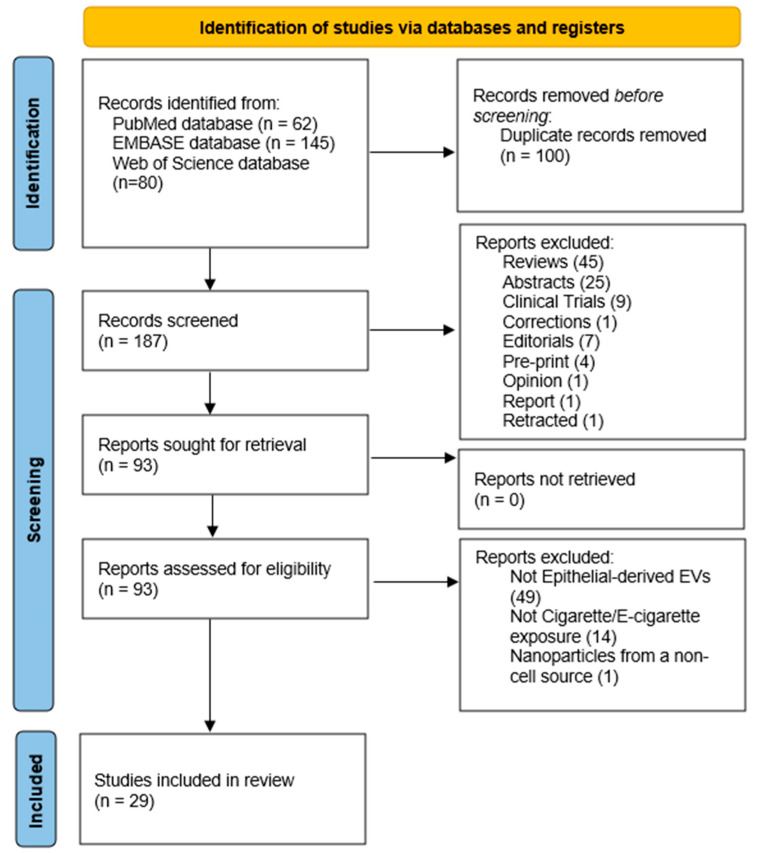
PRISMA 2020 flow chart detailing the process of article selection for this study.

Using these terms, the search was conducted on 17 December 2025, yielding a total of 287 articles: 62 from PubMed, 145 from Embase and 80 from Web of Science. Identified articles were imported into EndNote, where duplicates were removed. The remaining articles were initially screened to remove reviews and conference abstracts. Full texts of the remaining articles were then assessed by RS and independently by WB for eligibility for this systematic review according to the pre-defined inclusion and exclusion criteria (Table 1). A PRISMA 2020 diagram outlines the process of identifying and screening relevant articles for this systematic review (Figure 1).

### 2.2. Data Extraction and Quality Assessment

The search identified 29 papers, which provide information regarding the effect of cigarettes and e-cigarette products on epithelial-derived EVs. The robustness of the models used, as well as the research methodology and study design, were assessed, and the quality of these studies was scored in accordance with the criteria outlined in Table 2. Scoring of each paper was determined for each of the following sections: model used, cigarette smoke extract (CSE) and e-cigarette vapour condensate (ECVC) preparation, robustness of the model, sample size, EV isolation and EV characterization. This scoring system allows papers to receive multiple scores relating to their models, robustness or sample size, as some studies utilise multiple models. Consequently, the final score of each paper is presented as a percentage of the total marks. The aim of this scoring system was to provide a comprehensive assessment of current EV research in this area, with lower scores being associated with a lack of factors that could further strengthen the studies, such as a small sample size, and does not reflect their validity.

## 3. Results

### 3.1. PRISMA and Publication Selection

**Figure 2:**
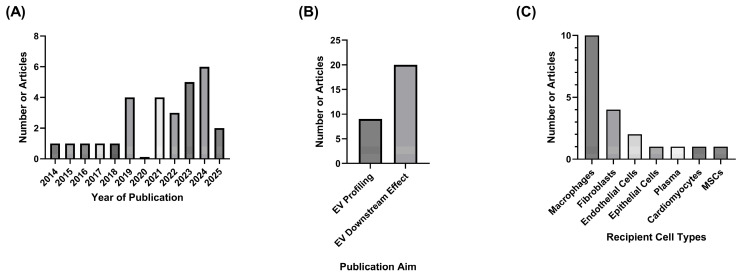
Metrics on publications identified for inclusion in the review. (**A**) Number of publications over time; (**B**) number of publications per publication aim; (**C**) number of publications concerning recipient cell types used to assess EV downstream effects.

In accordance with the inclusion and exclusion criteria, a total of 29 papers were identified in this review [40,41,42,43,44,45,46,47,48,49,50,51,52,53,54,55,56,57,58,59,60,61,62,63,64,65,66,67,68]. The earliest manuscript was published in 2014; however, 85% of the identified articles were published from 2019 onwards (Figure 2A). Analysis of the identified articles highlighted that research in this area focuses on either characterising epithelial-derived EVs secreted in response to cigarette and e-cigarette products, or on assessing the downstream effects these EVs might have on other cell types (Figure 2B), with the cell types examined highlighted in Figure 2C. Therefore, the results in this systematic review have been divided in accordance with the aims of the identified studies. Additionally, due to the variety of cell types used to assess downstream effects of EV, these results have been further divided into two categories depending on the recipient cell type: Epithelial-derived EVs interaction with Macrophages and Epithelial-derived EVs interaction with other cell types.

### 3.2. Characterising the Effect of CSE or ECVC on Lung Epithelial-Derived EVs

**Figure 3:**
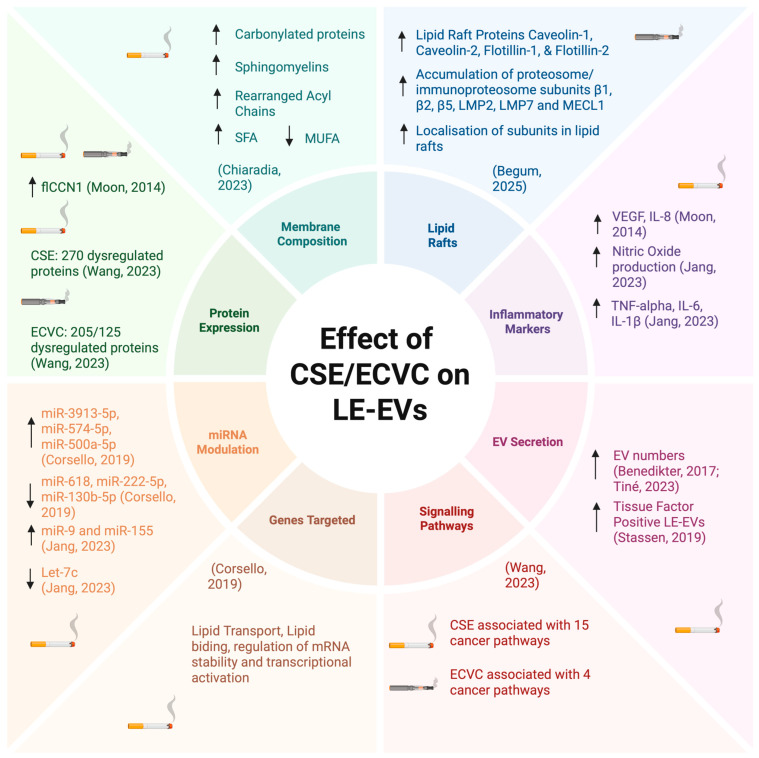
Key findings of the studies characterising the effect of CSE and ECVC on LE-EVs [40,41,42,43,44,45,46,47,48]. Saturated fatty acids (SFAs), mono-unsaturated fatty acids (MUFAs), full-length CCN1 (flCCN1). Created in https://BioRender.com.

Analysis of the 29 articles revealed that nine of these publications characterised the differences in lung epithelial-derived EVs (LE-EVs) (Supplementary Table S1) [40,41,42,43,44,45,46,47,48]. Figure 3 provides a detailed summary of the findings reported in these nine articles. Of the nine publications, seven described the impacts of CSE on the secretion and cargo of LE-EVs [40,41,42,43,45,47,48], one described the effect of ECVC with and without nicotine [44], and one assessed the effects of both traditional CSE and HTPs on LE-EV secretion and cargo [46]. Two papers reported that CSE promoted secretion of pro-inflammatory LE-EVs [40,48], with both papers associating CS-induced LE-EVs with COPD development. Two studies examined the impact of CSE on cell surface thiols and consequently on EV secretion [41,43]. In one study, CSE exposure depleted epithelial cells of cell surface thiols, increasing secretion of procoagulant LE-EVs [41]. In the other study, acrolein, an oxidative component present in CSE, increased LE-EV secretion and free thiols intracellularly, but cell surface thiols were depleted [43]. A study examined proteomic changes in exosomes secreted in response to CSE, unflavoured HTP and menthol flavoured HTP, and concluded that traditional cigarettes have a higher dysregulatory effect on exosomal proteins in comparison to e-cigarettes, as CSE exposure resulted in a higher number of differentially expressed exosomal proteins (DEEPs) in comparison to HTP exposure. Of these DEEPs, fifteen are involved in cancer pathways [46]. Two papers concluded that CSE exposure alters LE-EV’s lipid content [42,47]. One study reported that CSE exposure changes the phospholipid fatty acid composition of LE-EVs, altering EVs’ metabolic activity [47]. In the other paper, CSE exposure resulted in upregulation of miR-3913-5p, an mRNA predicted to be involved in lipid binding and transport, transcriptional activation, as well as regulation of mRNA stability [42]. One study also showed that ECVC induces transcription and translation of lipid raft-associated proteins [44].

With regards to quality scores, eight out of nine publications reporting information related to the effect of tobacco or nicotine products on LE-EVs utilised human models [40,41,42,43,44,45,46,47], with only one study relying on the use of a murine model, thus scoring lower in the model criteria [48]. The most common model used was cell culture, particularly human immortalised cell lines, reported in seven papers [40,41,42,43,44,46,47], with BEAS-2B being the most predominant cell line across studies. Only one paper implemented both animal and human models, as well as cell culture models using immortalised and primary human cells, hence receiving the highest model score; however, sample sizes were not defined for human and animal models, resulting in lower sample size scoring [40]. Only one paper assessed the presence of EVs in bronchoalveolar lavage fluid (BAL) patient samples in response to ECVC, being the only paper scored for ECVC preparation [45]. In general, studies received good human and murine robustness scores where applicable [40,45]. Although exposure was clearly defined in all papers, robustness of cell culture models received lower scores due to most studies utilising immortalised cell lines instead of primary cells [41,43,44,46,47,48], with the exception of one study that reported to use Human Primary Small Airway Epithelial cells from human donors and fully defined exposure, hence scoring the highest in this section [42]. Additionally, studies conducted on cellular models received, generally, good sample size scores [40,41,42,43,44,46,47], with the exception of one paper that did not report the sample size used [48]. In general, preparation of CSE was fully described [40,41,42,43,46,47], with the exception of one paper [48]. Furthermore, only three papers clearly stated the presence or absence of the cigarette filter, along with reporting the use of fresh CSE for exposure, thus receiving full scores [40,41,43,47]. Only one paper exposed cells to ECVC, clearly describing how this was prepared and disclosing the PG/VG ratio, thus receiving a good score [44].

Regarding EVs, the most common isolation method was sequential ultracentrifugation, used by seven studies [40,43,44,45,46,47,48]. One of these studies did not fully describe the isolation method [44]. One paper did not isolate the EVs prior to characterisation, resulting in a low score [41]. Only one paper reported using an ExoQuick EV precipitation kit, receiving the highest score [42]. Concerning EV characterisation, methods utilised varied between studies, with Western blots being used in six studies to profile EV surface proteins [40,42,43,46,47,48]. Only two papers reported using one method to characterise EVs, both using fluorescence-activated cell sorting (FACS) either to ensure EV origin [45] or to profile exosome markers [41]. To visualise EVs, three papers used transmission electron microscopy (TEM) [40,46,48], two used cryo-TEM [43,47] and one also included the use of Scanning Electron Microscopy (SEM) [47]. To analyse EV cargo, reverse transcription-quantitative polymerase chain reaction (RTq-PCR) was employed by two papers [42,46], and isobaric tag for relative and absolute quantitation (iTRAQ) by one paper [46]. Overall, no study scored below “poor”, with all papers profiling at least one EV biomarker and seven papers using several methodologies [40,42,43,44,46,47,48]. Indeed, the two papers with the lowest score characterised several biomarkers but only utilised one method to profile EVs [41,45].

All scores attained by the nine papers identified for this section are outlined in Supplementary Table S2. Five papers scored above 50% [40,42,43,46,47], three between 40 and 50% [41,44,45] and one paper scored lower than 30% [48]. The highest scoring was 67% due to the robust use of human, murine and cell line models [40], followed by 61% due to the variety of techniques used to isolate and comprehensively profile EVs [42].

### 3.3. Characterising Downstream Effects of LE-EVs

#### 3.3.1. On Macrophages

**Figure 4:**
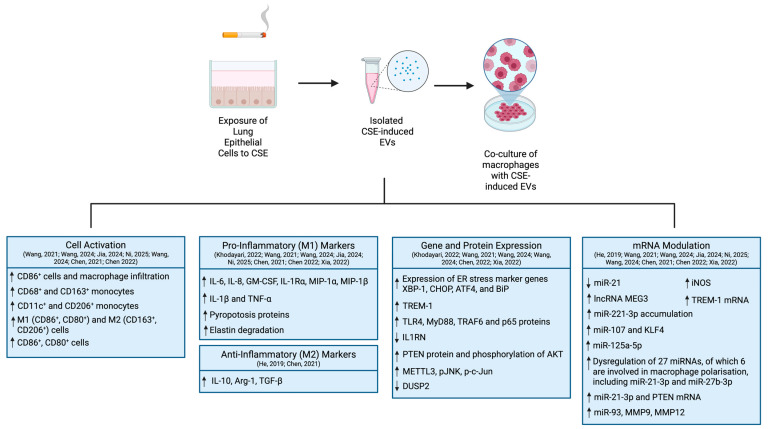
Key findings of the studies characterising the potential of CSE-induced LE-EVs to modulate macrophage responses [49,50,51,52,53,54,55,56,57,58]. Created in https://BioRender.com.

Ten articles reporting the potential of CSE-induced LE-EVs to modulate macrophage responses were identified (Supplementary Table S3) [49,50,51,52,53,54,55,56,57,58]. All articles identified used CSE as the stimulus to induce LE-EVs secretion. Figure 4 provides a detailed summary of the findings reported in these ten articles. Of these studies, eight described the effects of CSE-induced LE-EVs on M1/M2 polarisation, showing that CSE-induced LE-EVs could influence downstream immune responses [49,51,52,53,54,55,56,57]. One of these papers reported COPD patients to have higher exosomal miR-21 in total serum; however, levels of exosomal miR-21 found in CSE-induced LE-EVs were reduced, suggesting these can alleviate M2 macrophage polarisation [49]. Nonetheless, the reliability of the conclusions drawn from the cell culture model in this study raised questions regarding the controls and sample size. Six of the studies identified reported that CSE-induced LE-EVs promoted M1 polarisation and identified a wide variety of mRNAs (such as TREM-1 [51], lncRNA MEG3 [52], miR-221-3p [53], miR-107 [54], miR-125a-5p [55], miR-21-3p [56,57]) that become upregulated upon CSE exposure, with the potential to modulate monocyte differentiation towards M1 phenotype [51,52,53,54,55,57]. One paper also reported that miR-21-3p may be involved in macrophage polarisation; however, it concluded that CSE-induced exosomal miR-21-3p can promote secretion of both M1- and M2-associated cytokines, thus inducing both M1- and M2-macrophage polarisation. In addition to miR-21-3p, this study also revealed five other exosomal mRNAs that were altered in the presence of CSE, associated with pathways involved in the regulation of macrophage polarisation, namely miR-29a-3p and miR-27b-3p [56]. One article also revealed that exosomal miR-93 found in CSE-induced LE-EVs can influence emphysema progression through induction of elastin degradation [58]. Lastly, one paper studied the effects of CSE-induced LE-EVs in alpha-1 antitrypsin deficiency (AATD) macrophages, concluding that these EVs promote a pro-inflammatory environment [50].

Regarding quality scores, all ten studies looking at the potential of CSE-induced LE-EVs to modulate macrophage responses utilised cell culture models [49,50,51,52,53,54,55,56,57,58], of which eight used human cell cultures [49,50,53,54,55,56,57,58] and two relied on murine cell culture models [51,52]. A wide variety of human cell culture models were employed, with BEAS-2B being the most used cell line. Only two papers included more than one model, receiving higher model scores [49,58]. Coupled with human cell culture, one of these papers also implemented an animal model [58], and another study further included human samples, thus receiving the highest model score [49]. The robustness of studies varied with the models employed, with the robustness of cell culture models scoring lower [50,51,52,53,54,55,56,57] than those of animal and human models [49,58]. Only one study did not describe how the exposure was conducted, thus scoring the lowest [53]. The sample size was variable across studies, with seven studies utilising more than three repeats per experiment [50,52,53,54,56,57]; however, three did not define their sample size, thus not receiving a score [49,51,55]. Studies using animal models also varied in sample size, with one including ten animals per group [49], while the other did not define the number of animals per group [58]. Additionally, the only human study identified included five participants in both the healthy group and the COPD group [49]. In general, all groups described the method used to prepare CSE, with eight [49,50,51,52,54,55,56,58] also clearly stating if the cigarette filter was cut off and if the prepared extract was used within one hour of preparation, thus receiving high scores.

Concerning EVs, five out of the ten studies analysed isolated EVs using an ExoQuick precipitation kit [49,51,52,54,55], four isolated EVs through serial ultracentrifugation (UC) [50,56,57,58] and one implemented a sucrose cushion as part of the serial UC [53]. EV characterisation was comprehensive across studies, including three or more techniques, with all studies scoring “good” or better. Indeed, all ten studies used Western blot analysis to characterise two [49,55] or more EV biomarkers [50,51,52,53,54,56,57,58]. To confirm the presence of the isolated LE-EVs, nine papers included TEM analysis [49,50,51,52,54,55,56,57,58]. Nine studies used nanoparticle tracking analysis (NTA) to profile EV sizing, as well as RT-qPCR to characterise EV cargo [49,51,52,53,54,55,56,57,58]. Next-generation sequencing (NGS) was employed by one study to profile the miRNA composition of EVs [56]. Lastly, one study also included nano liquid chromatography coupled to tandem mass spectrometry (nano-LC-MS/MS) to characterise EV protein content [57]. Only one paper scored lower than 50%, receiving 42% [57], mainly due to the EV isolation method used (serial UC) and the robustness of the model. Generally, all studies received good scores, with the highest-scoring paper receiving 75% [49] due to the wide variety of models employed, good EV isolation and comprehensive EV characterisation. All scores are presented in Supplementary Table S4.

#### 3.3.2. On Other Cell Types

**Figure 5:**
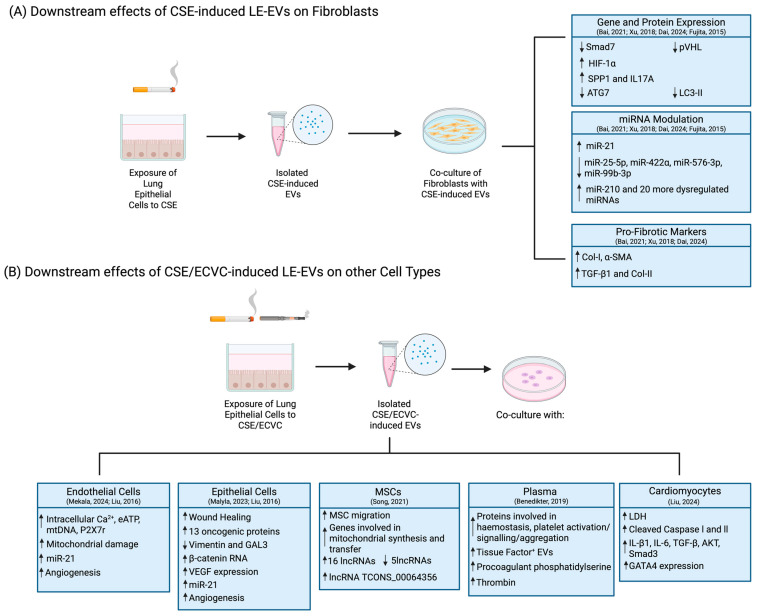
Key findings of the studies characterising the potential of CSE-induced LE-EVs to modulate downstream responses in other cell types [59,60,61,62,63,64,65,66,67,68]. Created in https://BioRender.com.

Of the 29 articles identified, ten characterised the potential of CSE/ECVC-induced LE-EVs to modulate downstream responses in other cell types (Supplementary Table S5) [59,60,61,62,63,64,65,66,67,68]. All ten publications reported that CSE-induced LE-EVs could influence cellular communication between epithelial cells and a range of cellular types, modulating downstream responses (Figure 5) [59,60,61,62,63,64,65,66,67,68]. Of these ten publications, four studied the potential of LE-EVs to crosstalk with fibroblasts [61,63,64,67], two with endothelial cells, of which one looked at brain endothelial cells [59], and the other studied both umbilical endothelial cells and bronchial epithelial cells [66]. Other recipient cell types included bronchial epithelial cells [60], MSCs [62], human plasma [65] and cardiomyocytes [68]. Five of the identified publications showed that LE-EVs were involved in the development of fibrosis [61,63,64,67,68]. Of these, four showed that CSE-induced LE-EVs can dysregulate signalling pathways in fibroblasts responsible for the development and exacerbation of pulmonary fibrosis [61,63,64,67]. One of these four publications studied the effect of e-cigarette exposure on cardiomyocytes and concluded that ECVC-induced LE-EVs promoted secretion of pro-fibrotic markers, aggravating cardiomyocyte pyroptosis [68]. Three papers showed that CSE-induced LE-EVs upregulated exosomal miR-21, associating this mRNA with the exacerbation of fibrosis [61,64] and angiogenesis [66]. Two papers studied the effects of LE-EVs in mitochondrial regulation [59,62]. One paper showed that EVs secreted in response to e-cigarette exposure, with nicotine, can induce mitochondrial stress, thus mitochondrial damage [59]. The other publication reported that CSE-induced LE-EVs can dysregulate genes involved in pathways responsible for the regulation of mitochondrial metabolism [62]. One study also revealed that CSE-induced LE-EVs promote the upregulation of genes involved in cancer pathways and observed that these EVs could promote wound healing when internalised by epithelial cells [60]. CSE-induced LE-EVs were also reported to upregulate the expression of prothrombotic proteins, suggesting CSE exposure alters EV’s cargo, promoting the secretion of procoagulant LE-EVs [65].

Of the ten articles assessing the potential of CSE/ECVC-induced-LE-EVs to modulate downstream responses of other cell types, nine utilised human cell cultures [59,60,61,63,64,65,66,67,68], one of which also included an animal model, receiving the highest score [68]. Several human cell culture models were used, with SV40-transformed normal human bronchial epithelial (HBE) cells being the most commonly used cell model. Only one paper relied entirely on murine cell culture models [62], thus scoring the lowest. Due to most studies employing human cell culture models, robustness was similar across studies [59,60,61,62,63,64,65,66], with only one paper not fully defining the exposure methods [68]. However, that was the only paper, including an animal model, with animal exposure fully defined and each animal group, including six animals [68]. Nine studies utilised more than three repeats per experiment [59,60,61,63,65,66,67,68]; nonetheless, two did not define their sample size and therefore did not receive a score [62,64]. Regarding stimuli preparation, five papers received the highest score for CSE preparation, clearly stating the methodology used and the presence or absence of the filter, and utilising the extract freshly made per experiment [61,62,63,64,65]. Most studies described the preparation method used, as well as the use of the extract within one hour of its preparation [60,61,63,64], with the exception of one paper that did not describe how the CSE was prepared, thus receiving no score [67]. Two papers used e-cigarette products, with one utilising e-cigarette liquid without it being vaped [59], thus not receiving a score, whereas the other study stated the PG/VG ratio but not the preparation method [68].

In regard to EVs, seven papers scored “good” in EV isolation [59,61,62,63,64,65,66], with five papers using an ExoQuick precipitation kit [59,61,63,64,66], one an exoEasy Maxi kit [62], and one implementing ultrafiltration prior to SEC [65]. Of the ten studies identified, eight characterised EVs extensively [59,60,62,63,64,65,66,67], with five papers employing varied techniques, as well as additional markers [60,62,63,64,65,67]. The presence of isolated EVs was confirmed using TEM in six papers [60,62,63,64,65,66]. Eight studies reported the use of NTA to profile EV sizes [59,60,61,62,63,64,65,67,68]. Seven studies performed Western blots to characterise two or more exosome biomarkers [59,62,63,64,66,67,68]. RT-qPCR was also used by five studies to comprehensively characterise EV cargo [60,62,63,64,66,67], with one paper performing a digital PCR [59]. Lastly, one study included tunable resistive pulse sensing (TRPS) to size EVs, and both nano-LC-MS/MS and nano-flow high-performance liquid chromatography (nanoflow HPLC) to characterise EV cargo [67]. Overall, all papers scored 50% or above, with two papers scoring 65% [63,65] due to the comprehensive and detailed EV isolation and characterisation conducted. Three papers scored 50%, either due to the exposure method [59], the use of a murine cell culture model [62] or lack of clarity regarding stimuli preparation [67]. All scores are outlined in Supplementary Table S6.

## 4. Discussion

The strain on healthcare systems globally due to tobacco product consumption is significant and well-documented [1,2]. Although the effects of conventional smoking are well studied, the underlying mechanisms by which the use of cigarettes, and potentially e-cigarettes, can disrupt intercellular communication within lung cells, resulting in chronic lung inflammation, are yet to be fully understood. Recent evidence suggests that EVs could play an important role in these early mechanisms and thus in the development of lung inflammation. This systematic review analyses the current research literature examining the impact of cigarette and e-cigarette products on LE-EVs, and how these toxic compounds can modulate EV composition, as well as downstream cellular responses.

In total, 29 papers studying the impact of cigarette or e-cigarette products on epithelial-derived EVs were identified [40,41,42,43,44,45,46,47,48,49,50,51,52,53,54,55,56,57,58,59,60,61,62,63,64,65,66,67,68]. Together, these papers showed that exposure of epithelial cells to tobacco products can influence EVs secreted by these cells. Some of the studies showed that CSE exposure leads to molecular changes in epithelial-derived EVs, suggesting these can be used as diagnostic tools [40,41,42,43,44,45,46,47,48], whereas others demonstrated that CSE- or ECVC-induced LE-EVs can transport and deliver aberrant cargo to neighbouring cells, ultimately influencing downstream cellular processes [49,50,51,52,53,54,55,56,57,58,59,60,61,62,63,64,65,66,67,68].

Twenty of these 29 papers reported that CSE- or ECVC-induced LE-EVs could promote cellular damage, inflammation or disease development, thus suggesting LE-EVs could be key players in the initial stages of lung inflammation and disease development [40,46,48,49,50,51,52,53,54,56,57,58,59,60,61,63,64,66,67,68]. One key mechanism identified in eight papers was the ability of LE-EVs to influence monocyte differentiation [49,51,52,53,54,55,56,57], with six studies reporting that CS-induced LE-EVs were capable of skewing macrophage polarisation towards an M1 phenotype, by promoting secretion of pro-inflammatory cytokines IL-1β, IL-6, TNF-α, and iNOS, while suppressing M2-associated cytokines IL-10 and Arg-1 [51,52,53,54,55,57]. In the lungs, exacerbated activation of M1 macrophages is associated with chronic lung inflammation and is a hallmark of inflammatory lung diseases such as COPD [69,70].

Exposure of epithelial cells to tobacco products also resulted in the upregulation of a wide variety of microRNAs (miRNAs) involved in different cell signalling mechanisms. miRNAs are non-coding RNAs involved in gene expression, regulating translation and transcription [71]. In some papers CSE exposure led to upregulation of miRNAs involved in pathways associated with lipid binding (miR-3913-5p [42]) or in the development of emphysema (miR-93 [58]), whereas others saw upregulation of miRNAs associated with macrophage polarisation (TREM-1 [51], lncRNA MEG3 [52], miR-221-3p [53], miR-107 [54], miR-125a-5p [55], and miR-21-3p [57]). One paper reported that miR-21-3p could skew macrophage polarisation towards both phenotypes [56]. A mechanism seen across three papers was the upregulation of miR-21, with evidence suggesting this miRNA promotes the development of fibrosis [61,63] and angiogenesis [66]. Interestingly, miRNA-21 has been shown to induce cell death in the lungs, exacerbating COPD symptoms [72] and involved in the progression of lung fibrosis [73]. Additionally, CSE-induced LE-EVs were also seen to increase the expression of fibrosis markers, either via downregulation of miR-422a expression in myofibroblasts [64] or via the upregulation of miR-210 in lung fibroblasts [67].

The presence of thiol-reactive compounds in CSE was identified as a mechanism responsible for altering EV composition [41,43]. In terms of EV’s diagnostic value, four papers reported that CS or ECA modulated LE-EV cargo, changing either encapsulated protein [40,46] or miRNA content [42,48], or EV membrane composition [41,47]. These papers showed that analysing the cargo and lipid membrane of EVs secreted by tobacco users could provide a new avenue for diagnosing early stages of lung inflammation. Additionally, increased epithelial EV numbers [45], as well as the accumulation of proteasome and immunoproteasomes subunits in response to ECA, were also reported as a possible biomarker for early lung inflammation [44].

Of the total 29 papers identified, only five received scores lower than 50% [41,44,45,48,57], with only one scoring lower than 30% that was published in 2023 [48]. Of these five papers, only one was published before 2022 [41], with one published in 2025 [44]. It is important to note that four of these papers had low scores regarding stimuli preparation [44,45,48,57], but all five papers utilised a cell culture model only, resulting in low robustness scores. Additionally, all five papers received low scores regarding EV isolation and characterisation methods.

Generally, transformed human cell lines were the most used cell culture model across all 29 papers, with only two studies including animal, human and cell culture models, resulting in these being the highest scoring papers (75% [49] and 67% [40]). Interestingly, both these papers were published before 2020, which might suggest a recent shift towards the use of cell culture models instead of animal models. The average score of papers analysing the effect of smoke and e-cigarette aerosol on the content and composition of epithelial-derived EVs [40,41,42,43,44,45,46,47,48] was 50.5%, 6% lower than the average of studies looking at the effects of EVs on macrophages (56.3% [49,50,51,52,53,54,55,56,57,58]), and 5% lower than the average of those using other cell types as recipient cells (55.2% [59,60,61,62,63,64,65,66,67,68]). The lower average of studies profiling EV responses can be explained by the prevalence of both poor isolation techniques (78% of these studies scored poor or lower [40,41,43,44,45,46,48]) and not fully defined stimuli preparation (seen in 50.5% of these papers [42,44,45,46,48]). Indeed, EV isolation and characterisation method scores were higher in studies assessing downstream effects of LE-EVs [49,50,51,52,53,54,55,56,57,58,59,60,61,62,63,64,65,66,68], with all the papers identified in this systematic review assessing the impact of epithelial EVs on macrophage responses scoring 75% or above regarding their characterisation techniques [49,50,51,52,53,54,55,56,57,58]. Interestingly, 70% of the papers studying the effect of epithelial EVs on other cell types scored the highest in the EV isolation [59,61,62,63,64,65,66], with another 80% scoring 75% or above regarding their characterisation techniques [59,60,62,63,64,65,66,67]. The adoption of high-purity isolation methods, coupled with more detailed and comprehensive characterisation techniques, is essential to allow for accurate and reliable conclusions, thus granting studies higher scores. Future research should continue to ensure the adoption of such techniques, minimising EV damage, as well as the introduction of impurities. Additionally, the implementation of methodologies that allow for the identification of sub-populations of epithelial-derived EVs could prove to be a potential alternative with diagnostic value. This could allow for the identification of specific EV populations secreted in response to cigarette and e-cigarette exposure, presenting altered surface markers.

EVs provide a novel avenue to better understand disease development and progression. Due to their ability to retain their parental cell properties, these vesicles can also elucidate the pathological state of the parent cell, thus providing the potential for new diagnostic tools [32,33]. Because of these properties, interest in EVs in the context of lung injury is ever-growing, particularly focusing on the role that EVs play in lung pathologies [74]. In the lungs, EVs have been associated with the development of tissue injury, disease, and chronic inflammation [32,35,36,74]. Current research investigating the effect of CS on EVs not only examines epithelial-derived EVs as reviewed here but also covers an extensive range of parent cell types, including EVs derived from plasma [75] and dendritic cells [76].

Most of the papers identified in this systematic review used cell culture models instead of human samples or animal models, relying on cell monolayers (two-dimensional models). Moving forward, the use of multi-cell-type three-dimensional (3D) models would be beneficial, particularly coupled with air-liquid-interface (ALI) culture. This cell culture model would enable cell differentiation, better mimicking the lung epithelial barrier [77]. This would improve the robustness of cell culture models, as it results in a more biologically relevant model. Additionally, cell exposure to stimuli was mainly conducted by submerging cell cultures in CSE. This was emphasised by the fact that of the 29 papers identified, only three assessed the effect of e-cigarettes [44,59,68] and one of HTPs [46]. It is worth noting that preparation and exposure of cell cultures to e-cigarette products varied significantly across these papers, with one paper adding e-liquid directly to cells [59], one adding ECA-conditioned media to the cells [44], and one exposing an animal model to ECA directly [68]. Adoption and standardisation of methods that allow the aerosolisation of stimuli to better reflect in vivo conditions, such as exposure systems, would be beneficial and allow for a better comparison across papers [78]. Finally, taking into account the rapid uptake of e-cigarettes as an alternative to conventional cigarettes over the past decade, it is of the utmost importance to understand the impacts that these devices might have on lung inflammation. Indeed, in the United Kingdom, the number of people who use e-cigarettes has just overtaken the number of people who smoke conventional cigarettes, emphasising the need for this research [79]. Therefore, future research must extend to e-cigarettes, as well as HTPs, as these are increasing in prevalence.

## 5. Conclusions

This systematic review analysed the current research investigating the impacts of cigarette and e-cigarette exposure on LE-EVs. The articles reviewed suggest that tobacco products can influence EV secretion from epithelial cells, as well as transport and deliver aberrant cargo to recipient cells. The mechanisms identified by which tobacco products could modulate EV secretion included altering EV composition, changing the cargo and the lipid-membrane of EVs, as well as increasing the number of EVs secreted. In turn, altered EVs were found to modulate monocyte differentiation by promoting the secretion of cytokines associated with M1/M2 macrophage polarisation, as well as modulating the expression of genes in the recipient cell. The lack of appropriate models, coupled with the limited number of published papers studying e-cigarettes, highlights the need for further research in this field. As e-cigarettes become more popular among young people, it is essential to advance the current understanding regarding the impact of such devices on LE-EVs.

## Figures and Tables

**Table 1:** The inclusion and exclusion criteria used to determine eligibility for this study.

Inclusion	Exclusion
Research that includes cigarette and e-cigarette smoke exposure	Non-original research papers, e.g., reviews, editorials, notes, case reports, etc.
Research containing lung epithelial-derived EVs	Conference abstracts
Research involving isolation, identification or production of EVs or their contents (DNA, miRNA or protein)	Pre-prints
Clearly described EV isolation methods	Research involving microparticles but not extracellular vesicles from a cellular source
Clinical data from current smokers	Non-epithelial-derived EVs
Experimental data	Research involving lung damage or cancer, but not smoking related
Human models	Research involving non-cigarette/e-cigarette smoke exposure, e.g., woodsmoke or waterpipe smoke
Animal data	Clinical data involving ex-smokers

**Table 2:** Criteria used for quality assessment.

Category	Scoring Criteria
Model: If multiple models are used, a combined score is provided by:	Cell culture (in vitro—murine) (1) Animal model (in vivo) (2) Cell culture (in vitro—human) (3) Human (ex vivo) (4)
Method used for CSE preparation Summative score of all points	Methods used for CSE preparation are clearly described (1) CSE is used within an hour of being prepared (1) Clearly stated if cigarettes were burnt with or without cigarette filter (1)
Method used for ECVC preparation: Summative score of all points	Methods used to generate ECVC clearly described (1) PG/VG ratio clearly defined (1) ECVC used shortly after prepared (1)
Robustness of model: If multiple models are used, a combined score is provided by:	Human model (ex vivo): Cigarette/e-cig smokers or no-smokers were sought from a clinical setting (1) Cigarette/e-cig smokers were sought from a clinical setting with a healthy control group (2)
	Animal model (in vivo): Animal exposure to CSE/ECVC is partially defined (discloses some but NOT all the following: exposure method, dosing, and duration) (1) Animal exposure to CSE/ECVC is fully defined, outlining (exposure method, dosing, and duration) (2)
	Cell culture (in vitro): Transformed cell line using partially defined exposures (exposure method, dosing, and duration) (1) Transformed cell line using fully defined exposures (exposure method, dosing, and duration) (2) Primary Cells using partially defined exposures (discloses some but NOT all the following: exposure method, dosing, and duration) (3) Primary cells using fully defined exposures (exposure method, dosing, and duration) (4)
Sample size: If multiple models are used, a combined score is provided by:	Human model (ex vivo): Number of participants not defined (0) 5 or fewer participants per group (1) 6 to 11 participants per group (2) 12 or more participants per group (3)
	Murine model (in vivo): Number of animals not defined (0) 5 or fewer animals per group (1) 6 to 11 animals per group (2) 12 or more animals per group (3)
	Cell culture (in vitro): n not specified (0) n < 3 (1) n ≥ 3 (2)
EV isolation	No isolation—precipitation only techniques. Studies looking direct in a liquid with no isolation applied (usually done on very low volume samples). (0) Poor—ultra centrifugation at one speed or serial UC without sucrose cushion. (1) Fair—size exclusion chromatography (without a precipitation or concentration step) or immuno—capture beads for investigation of non-specific populations. (2) Good—size exclusion chromatography with the use of a specific isolation method, such as exosome EV isolation, purification kits or immuno-capture beads utilising a unique marker. (3)
EV characterisation	No characterisation—No attempt made to profile or characterise EVs or exosomes. (0) Poor—use of just one characterisation technique (quantification, sizing, biomarkers, cargo analysis) (1) Fair—the use of multiple complimentary characterisation techniques and at least 1 biomarker. (2) Good—everything mention prior, as well as appropriate controls (e.g., robust profiling of culture conditions, such as media, inclusion of positive and negative controls such as those recommended by MISEV2023), as well as characterisation of single EVs (3)

## Data Availability

The original contributions presented in the study are included in the article; further inquiries can be directed to the corresponding author.

## Supplementary Materials

The following supporting information can be downloaded at: https://www.mdpi.com/article/10.3390/ijms27062787/s1.

## Abbreviations

The following abbreviations are used in this manuscript:

EVs	Extracellular Vesicles
LE-EVs	Lung Epithelial-derived Extracellular Vesicles
CS	Cigarette Smoke
CSE	Cigarette Smoke Extract
ECA	Electronic Cigarette Aerosol
ECVC	Electronic Cigarette Vapour Condensate
HTP	Heated Tobacco products
ENDS	Electronic Nicotine Delivery Systems
PG	Propylene Glycol
VG	Vegetable Glycerine
COPD	Chronic Obstructive Pulmonary Disease
AJ	Adheren Junction
TJ	Tight Junction
MSCs	Mesenchymal Stem Cells
DEEPs	Differentials Expressed Exosomal Proteins
HBE	Human Bronchial Epithelial
FACS	Fluorescent-Activated Cell Sorting
TEM	Transmission Electron Microscopy
SEM	Scanning Electron Microscopy
RTq-PCR	Reverse Transcription-quantitative Polymerase Chain Reaction
UC	Ultracentifucation
SEC	Size Exclusion Chromatography
iTRAQ	Isobaric Tag for Relative and Absolute Quantitation
