# Supplementary material for: The Effect of Cigarettes and E-Cigarettes on Epithelial-Derived Extracellular Vesicles: A Systematic Review
Source: Int J Mol Sci. 2026 Mar 19;27(6):2787. doi: 10.3390/ijms27062787 (PMC13026786; doi:10.3390/ijms27062787)
Supplement: Supplementary file 1 [file ijms-27-02787-s001.zip › ijms-4162300-supplementary.pdf]

Supplementary Information

Table S1. Studies characterising the effect of CSE or ECVC on LE-**EVs.**

First Author and Year	Model	Isolation	Characterisation	Reported Findings
Moon et al., 2014 [40]	Human patients, human cell line (BEAS-2B), primary cells, mouse model (C57BL/6)	Serial Ultracentrifugation 100,000× g 2h, 100,000× g 2h	TEM and Western Blot	CSE-induced epithelial-derived exosomes encapsulated high levels of flCCN1. Increased extracellular flCCN1 induced the release of IL-8 and VEGF.
Stassen et al., 2019 [41]	Human cell line (BEAS-2B)	No isolation	FACS	CSE exposure resulted in the secretion of procoagulant Tissue Factor+ LE-EVs via thiol reactive species. Treatment with the antioxidant NAC inhibited CS-induced EV release.
Corsello et al., 2019 [42]	Human Primary Small Airway Epithelia cells	Precipitation - EXOQuick Kit and SEC	NTA, Western Blot, NGS and RT-PCR	CSE-induced LE-EVs were significantly enriched in miRNAs. The most upregulated miRNAS (miR-3913-5p) is predicted to be involved in lipid transport, lipid binding and transcriptional activation.
Benedikter et al., 2017 [43]	Human cell line (BEAS-2B)	Serial Ultracentrifugation: 300 x g 10min, 5,000 x g 10min, 117,734 x g 2:30h	FACS, Tunable resistive pulse sensing (TRPS), cryo-TEM and Western Blot	CSE induced LE-EVs secretion in response to thiol-reactive compounds (acrolein). CSE and acrolein exposure increased intracellular free thiols. Blocking of third disulfide exchange reactions on cell surface promoted exosome secretion.

Begum et al., 2025 [44]	Human cell line (A549)	The harvested cell culture media was subjected to centrifugation. To the resulting cell-free culture media, 0.5 volumes of the total exosome isolation reagent was added. The samples were then incubated overnight and then centrifuged for 1 h.	Immunoblotting, dot blot or hybrid ELISA	ECVC exposure increased expression and accumulation of constitutive and immunoproteasome subunits-β1, β2, β5, LMP2, LMP7 and MECL1 in addition to Caveolin-1, Caveolin-2, Flotillin-1, and/or Flotillin-2 in LE-EVs.
Tinè et al., 2023 [45]	Human patients	Bal: Serial Ultracentrifugation: 350 x g 10min, 10,000 x g 30min Blood: 1,500g x 15 min, 13,000g x 2 min	FACS	LE-EVs isolated from human patients did not differ in number between smokers with and without COPD but were significantly increased in comparison to non-smokers.
Wang et al., 2023 [46]	Human cell line (16HBE cells)	Serial Ultracentrifugation: 500× g 10min, 12,000× g 20min 12,000× g 20min, 120,000× g 1h, 120,000× g h	TEM, RT-PCR, Malvern laser particle size analyzer, iTRAQ analysis and Western Blot (CD9, CD63)	LE-EVs secreted from CSE exposed cells had the highest number of DEEPs in comparison to unflavoured and flavoured HTP-derived exosomes. Proteomic analysis of isolated LE-EEs showed changes in exosomal protein expression of a wide variety of proteins mainly involved in key pathways associated with cancer development, particularly in response to CSE exposure.
Chiaradia et al., 2023 [47]	Human cell line (BEAS-2B)	Serial Ultracentrifugation: 300× g 10min, 2,000× g 10min, 10,000× g 30min, 100,000× g 70min, 100,000× g 70min. “In some cases, EVs were further purified using SEC”	SEM, NTA, cryo-TEM and Western Blot	CSE exposure resulted in LE-EVs containing high content of carbonylated proteins, and altered the structure of EV membrane phospholipids, significantly increasing palmitic and arachidic acids, while reducing

				sapienic and oleic acids. CSE induced a metabolic shift, resulting in higher SFA and lower MUFA, increasing the SFA/MUFA ration in CSE-induced LE-EVs.
Jang et al., 2023 [48]	Murine cell line (MLE-12 cells) and mouse model (C57BL/6)	Serial Ultracentrifugation: 2,000× g 10min, 10,000× g 30min, 75,000× g	FluoroCet Ultrasensitive Exosome Quantitation Assay, microRNA assay, NTA, TEM and Western Blot	PTD-FGF2 treatment inhibited NO, intracellular ROS and pro-inflammatory cytokine production via inhibition of JNK/ERK and p38 MAPK pathway in CSE-exposed epithelial cells. PTD-FGF2 treatment altered exosomal miRNA expression, increasing the secretion of anti-inflammatory exosomes with upregulated Let-7c expression, and downregulated miR-9 and miR-155 expression.

Table S2. Scoring of papers characterising the effect of CSE or ECVC in LE- EVs profile.

First Author and Year	Model (n/10)	Robustness of Model			Sample Size			Preparation of Exposure (n/3)	EV isolation (n/3)	EV Characterisation (n/4)	Bias Score
		Murine Model (n/2)	Cell Culture (n/4)	Human Studies (n/2)	Murine Model (n/3)	Cell culture (n/2)	Human Studies (n/3)				
Moon et al., 2014 [40]	Human patients (4), Human Cell culture (3), Animal model (2)	CSE exposure fully defined (2)	Transformed cell line (1) and primary cells (3), partially defined exposure	From clinical setting with healthy control group (2)	Number of animals per group not defined (0)	2 or 3 repeats per experiment (1)	Number of patients per group not defined (0)	CSE preparation is clearly described (1) absence of the filter is clearly stated (1) and used within 1 hour of being prepared (1)	Serial UC (1)	Multiple complimentary techniques (2)	66.66 %
Stassen et al., 2019 [41]	Human Cell culture (3)		Transformed cell line, fully defined exposure (2)			More than 3 repeats per experiment (2)		CSE preparation is clearly described (1), absen	No isolation (0)	One method utilized (1)	42.31 %

				ce of the filter is clearly stated (1) and used within 1 hour of being prepared (1)			
Corsello et al., 2019 [42]	Human Cell culture (3)	Exposure of primary cells fully defined (4)	3 or 4 repeats per experiment (2)	CSE preparation is clearly described (1)	Precipitation—EXOQuick-TC (3)	Multiple complementary techniques and suitable controls (3)	61.54 %
Benedikter et al., 2017 [43]	Human Cell culture (3)	Transformed cell line, fully defined exposure (2)	3 or 4 repeats per experiment (2)	CSE preparation is clearly described (1), absence of the filter is clearly stated (1) and used within 1 hour of being prepared (1)	Serial UC (1)	Multiple complementary techniques and suitable controls (3)	53.85 %

Begum et al., 2025 [44]	Human Cell culture (3)	Transformed cell line, fully defined exposure (2)		More than 3 repeats per experiment (2)		ECVC preparation is clearly described (1) and PG/VG ratio clearly stated (1)	UC poorly defined (1)	Multiple complimentary techniques (2)	46.15%
Tinè et al., 2023 [45]	Human patients (4)		From clinical setting with healthy control group (2)		9 to 11 participants per group (2)		Serial UC (1)	One method utilized (1)	45.45%
Wang et al., 2023 [46]	Human Cell culture (3)	Transformed cell line, fully defined exposure (2)		More than 3 repeats per experiment (2)		CSE and HTP smoke extract preparation is clearly described (1)	Serial UC (1)	Multiple complementary techniques, suitable controls and additional biomarkers (4)	53.85%
Chiaradia et al., 2023 [47]	Human Cell cult	Transformed cell line, fully		More than 3 repeats per experi		CSE preparation is clearl	Serial UC, "in some cases SEC" (2)	Multiple complementary techniques and	57.69%

	ure (3)	defined exposure (2)		ment (2)	y described (1), absence of the filter is clearly stated (1) and used within 1 hour of being prepared (1)		suitable controls (3)	
Jang et al., 2023 [48]	Murine Cell culture (1), Animal model (2)	Transformed cell line, fully defined exposure (2)	Number of animals per group not defined (0)	Number or repeats not defined (0)	CSE preparation not described (0)	Serial UC (1)	Multiple complimentary techniques (2)	27.59%

Table S3. Studies describing the potential of CSE-induced LE-EVs to modulate macrophage responses.

First Author and Year	Model	Recipient Cells	Isolation	Characterisation	Reported Findings
He et al., 2019 [49]	Human patients, mouse model (C57BL/6), human cell line (BEAS-2B)	Macrophages (THP-1)	Precipitation - EXOQuick Kit	NTA, TEM, Western Blot, RT-qPCR	COPD patients have high levels of exosomal miR-21 in serum. COPD mice expressed high levels of mesenchymal markers in their lungs, suggesting EMT occurred. CSE reduced levels of miR-21 in LE-EVs suggesting inhibition of M2-polarisation.
Khodayari et al., 2022 [50]	Human Primary Small Airway Epithelial cells	Serial Ultracentrifugation: 10,000× g 30min, 118,000× g 70min, 118,000× g 70min	TEM, qRT-PCR and Western Blot		Internalisation of CSE-induced LE-EVs by AATD macrophages results in an increased pro-inflammatory macrophage. In AATD macrophages CSE-induced EVs, increased secretion of f pro-inflammatory cytokines, as well as AAT polymers.

Wang et al., 2021 [51]	Murine cell line (MAECs)	Precipitation - EXOQuick Kit	TEM, NTA and Western Blot	CSE-induced LE-EVS promoted M1 macrophage polarisation in vitro and in vivo, increased CD86+ macrophages, and increased macrophage expression of M1 markers via upregulation of TREM-1 mRNA.
Wang et al., 2024 [52]	Murine cell line (MAECs)	Precipitation - EXOQuick Kit	TEM, NTA and Western Blot, RT-qPCR	CSE-treated MAECs-derived exosomal lncRNA MEG3 exacerbates lung injury, M1 macrophage polarisation, and pyroptosis in COPD mice, via the SPI1/METTL3/TREM-1 axis.
Jia et al., 2024 [53]	Human cell line (HBE-16 and Beas-2B)	Sucrose gradient density (using 3 different densities) ultracentrifugation: 100,000× g 2:30h	NTA, Western Blot, RT-PCR, TEM	CSE-induced LE-EVs promoted a shift in macrophage phenotype towards M1 macrophage polarisation. CSE-induced EVs contained high levels of miR-221-3p which suppressed SOC3 mRNA expression in macrophages, while promoting phosphorylation of STAT3.
Ni et al., 2025 [54]	Human cell line (BEAS-2B)	Precipitation - EXOQuick Kit	NTA, TEM and Western Blot, RT-qPCR	CSE-induced LE-EVs internalised by macrophages increased CD11c+ and CD206+ macrophages and significantly induced the secretion of pro-inflammatory markers. CS-E-

				induced LE-EVs contained high levels of miR-107 which activated the Nf-kb signalling pathway and promoted a M1 phenotype.
Wang et al., 2024 [55]	Human cell line (HBE and Beas-2B)	Precipitation - EXOQuick Kit	TEM, NTA, qPCR and Western Blot	Exosonal miR-125a-5p reduced IL1RN expression in macrophages and kidney cells, and increased the expression of TLR4, MyD88, TRAF6 and p65 proteins in macrophages. CS-induced LE-exosomal miR-125a-5p overexpression promoted M1 macrophage polarisation by directly targeting and inhibiting IL1RN protein expression and subsequently activating the MyD88/TRAF6/TLR/NF-kb signalling pathway.
Chen et al., 2021 [56]	Human cell line (BEAS-2B)	Serial Ultracentrifugation: 300× g 10min, 2,000× g 10min, 10,000× g 30min, 100,000× g 70min	TEM, NTA, NGS, RT-qPCR, and Western Blot	CS-induced LE-EVs induced both M1 and M2 polarisation, as well as secretion of both M1 and M2 related cytokines. CSE exposure resulted in alterations in LE-EVs mRNAs involved in key pathways associated with the regulation of macrophage polarisation.

Chen et al., 2022 [57]	Human cell line (BEAS-2B)	Serial Ultracentrifugation: 300× g 10min, 2,000× g 10min, 10,000× g 30min, 100,000× g 70min	TEM, NTA, nano-LC-MS/MS, RT-qPCR and Western Blot	Naringenin suppressed M1 macrophage polarisation induced by CSE-induced LE-EVs through the downregulation of exosome miR-21-3p.
Xia et al., 2022 [58]	Mousse model (BALB/c) and human cell line (HBE)	Serial Ultracentrifugation: 300× g 10min, 2,000× g 30min, 12,000× g 45min, 100,000× g 120min, 100,000× g 70min, 100,000× g 70min	NTA, TEM, Western Blot (CD9, CD63, CD81), RT-qPCR	CSE-induced LE-EVs encapsulated high levels of miR-93, which suppresses DUSP2. Suppression of DUSP2 in macrophages triggers the JNK pathway ultimately promoting the progression of emphysema.

Table S4. Scoring of papers studying the potential of CSE-induced LE-EVs to modulate macrophage responses.

First Author and Year	Model (n/10)	Robustness of Model			Sample Size			Preparation of Exposure (n/3)	EV isolation (n/3)	EV Characterisation (n/4)	Bias Score
		Murine Model (n/2)	Cell Culture (n/4)	Human Studies (n/2)	Murine Model (n/3)	Cell culture (n/2)	Human Studies (n/3)				
He et al., 2019 [49]	Human patients (4), Human Cell culture (3), Animal model (2)	CSE exposure fully defined (2)	Transformed cell line partially defined exposure (1)	From clinical setting with healthy control group (2)	10 animals per group (2)	Number of repeats not specified (0)	5 participants per group (1)	CSE preparation is clearly described (1), absence of the filter is clearly stated (1) and used within 1 hour of being prepared (1)	Precipitation—EXO Quick-TC (3)	Multiple complementary techniques, suitable controls and additional biomarkers (4)	75.00%
Khodayari et al., 2022 [50]	Human Cell culture (3)		Transformed cell line, fully defined exposure (2)			3 or more repeats per experiment (2)		CSE preparation is clearly described (1), absence of the filter is clearly stated (1) and used within 1 hour of being prepared (1)	Serial UC (1)	Multiple complementary techniques and suitable controls (3)	53.85%

Wang et al., 2021 [51]	Murine Cell culture (1)	Transformed cell line, fully defined exposure (2)	Number of repeats not specified (0)	CSE preparation is clearly described (1), absence of the filter is clearly stated (1) and used within 1 hour of being prepared (1)	Precipitation—EXO Quick-TC (3)	Multiple complementary techniques, suitable controls and additional biomarkers (4)	50.00%
Wang et al., 2024 [52]	Murine Cell culture (1)	Transformed cell line, fully defined exposure (2)	3 or more repeats per experiment (2)	CSE preparation is clearly described (1), absence of the filter is clearly stated (1) and used within 1 hour of being prepared (1)	Precipitation—EXO Quick-TC (3)	Multiple complementary techniques, suitable controls and additional biomarkers (4)	57.69%
Jia et al., 2024 [53]	Human Cell culture (3)	Transformed Cell Line not defined (0)	4 or more repeats per experiment (2)	CSE preparation is clearly described (1) and used within 1 hour of being prepared (1)	Sucrose Cushioned UC (2)	Multiple complementary techniques, suitable controls and additional biomar	50.00%

						kers (4)	
Ni et al., 2025 [54]	Human Cell culture (3)	Transformed cell line, fully defined exposure (2)	3 repeats per experiment (2)	CSE preparation is clearly described (1), absence of the filter is clearly stated (1) and used within 1 hour of being prepared (1)	Precipitation—EXO Quick-TC (3)	Multiple complementary techniques, suitable controls and additional biomarkers (4)	65.38%
Wang et al., 2024 [55]	Human Cell culture (3)	Transformed cell line, fully defined exposure (2)	Number of repeats not specified (0)	CSE preparation is clearly described (1), absence of the filter is clearly stated (1) and used within 1 hour of being prepared (1)	Precipitation—EXO Quick-TC (3)	Multiple complementary techniques and suitable controls (3)	53.85%

Chen et al., 2021 [56]	Human Cell culture (3)		Transformed cell line, fully defined exposure (2)		3 or more repeats per experiment (2)	CSE preparation is clearly described (1), absence of the filter is clearly stated (1) and used within 1 hour of being prepared (1)	Serial UC (1)	Multiple complementary techniques and suitable controls (3)	53.85%
Chen et al., 2022 [57]	Human Cell culture (3)		Transformed cell line partially defined exposure (1)		3 or more repeats per experiment (2)	CSE preparation is clearly described (1)	Serial UC (1)	Multiple complementary techniques and suitable controls (3)	42.31%
Xia et al., 2022 [58]	Human Cell culture (3), Animal model (2)	CSE exposure fully defined (2)	Transformed cell line, fully defined exposure (2)	Number of animals per group not defined (0)	3 or more repeats per experiment (2)	CSE preparation is clearly described (1), absence of the filter is clearly stated (1) and used within 1 hour of being prepared (1)	Serial UC (1)	Multiple complementary techniques, suitable controls and additional biomarkers (4)	61.3 %

Table S5. Studies describing the potential of CSE-induced LE-EVs to modulate downstream responses in other cell types.

First Author and Year	Model	Recipient Cells	Isolation	Characterisation	Reported Findings
Mekala et at., 2024 [59]	Human cell line (HPAEpiC)	Human Brain Microvascular Endothelial Cells (HBMVEC)	Precipitation - EXOQuick Kit	NTA, Western Blot, digital PCR, ATP detection kit and FACS	E-cigarette containing 1.8% nicotine increase secretion and larger sized LE-EVs. E-cig (1.8%)-induced LE-EVs showed higher levels of eATP, mtDNA and P2X7r. Culture of endothelial cells with E-cig (1.8%)-induced-LE-EVs increased intracellular levels of Ca2+, increasing mitochondrial stress.
Malyla et at., 2023 [60]	Human cell line (16HBE14o)	Human epithelial cell line (16HBE14o)	Serial Ultracentrifugation: 18,000× g 90min, 2,000× g 2min, 18,000× g 90min	FACS, TEM, dynamic light scattering (DLS), protein array, RT-PCR.	CSE induced secretion of LE-EVs rich in b-catenin RNA. Exosomal b-catenin RNA upregulated gene expression (of WNT/β-catenin genes) in recipient cells. CSE-induced-LE-EVs upregulated 13 oncology genes and b-catenin RNA expression in recipient cells. CSE-EVs also promoted wound healing.

Bai et at., 2021 [61]	Human cell line (SV40-transformed normal Human bronchial epithelial cell (HBE) lines)	Human fibroblasts (MRC-5)	Precipitation - EXOQuick Kit	NTA	CS downregulated circRNA_002634, while increased levels of miR-21 in epithelial cells and EVs. In fibroblasts, exosomal miR-21 increased the expression of fibrosis markers Smad3 and TGFB1, as well as pro-fibrosis cytokines, modulating the activation of TGFb1/Smad3 signalling pathway.
Song et at., 2021 [62]	Murine cell line (AEC-II)	Murine MSCs	exoEasy Maxi Kit	NTA, TEM, Western Blot, RT-qPCR	CSE dysregulated 21 exosomal lncRNAs; 9 predicted to be involved in the regulation of membrane potential, gene expression, utero embryonic development, and in the regulation of mitochondrial functions. Overexpression of one of these lncRNAs (TCONS_00064356) promoted migration of MSCs and upregulation of genes involved in mitochondrial synthesis and transfer.

Xu et at., 2018 [63]	Human cell line (SV40-transformed normal HBE cell lines)	Human fibroblasts (MRC-5)	Precipitation - EXOQuick Kit	NTA, TEM, qRT-PCR and Western Blot	Cigarette smoke up-regulated LE-Exosomal miR-21, which promoted myofibroblast differentiation through upregulating fibrosis markers a-SMA and COL I and increasing HIFa activity.
Dai et at., 2024 [64]	Human cell line (SV40-transformed normal HBE cell lines)	Human fibroblasts (MRC-5)	Precipitation - EXOQuick Kit	NTA, TEM, qRT-PCR, Western Blot	CSE-induced LE-EVs expressed reduced levels of four miRNAs, miR-422a being the lowest. In fibroblasts, CSE-induced LE-EVs reduced the expression of miR-422a and increased the expression of fibrosis markers SPP1, IL-17A, as well as a-SMA and COL I.
Benedikter et at., 2019 [65]	Human cell line (BEAS-2B)	Human samples (plasma)	Ultrafiltration and Sepharose CL-4B SEC	cryo-TEM, TRPS, nanoflow HPLC, nano-LC–MS/MS, FACS	CSE modulated LE-EV protein expression, resulting in increased CD81+ and CD9+ EVs, as well as TF+ EVs and EVs with 154 upregulated prothrombotic proteins, which result in increased PS and consequentially promoting thrombin production.
Liu et at., 2016 [66]	Human cell line (SV40-transforme	Primary human endothelial cells	Precipitation - EXOQuick Kit	TEM, Western Blot, RT-qPCR	CSE exposure of epithelial cells upregulated miR-21 expression

	d normal HBE cell lines)	(HUVECs) and HBE cells			through STAT3 activation, resulting in increased levels of exosomal miR-21. In endothelial cells, exosomal miR-21 upregulated VEGF expression, promoting angiogenesis through modulation of STAT3/VEGF activation.
Fujita et al., 2015 [67]	Primary Human Bronchial Epithelial Cells and Human cell line (BEAS-2B)	Primary human lung fibroblasts and human fibroblasts (MRC-5)	Serial Ultracentrifugation: 35,000 rpm 70 min, 35,000rpm 70 min,	NTA, Western Blot, EM, mRNA microarray, RT-qPCR	LE-EVs secreted in response to CSE resulted in altered expression of 8 miRNAs (let-7a, let-7b, let-7c, let-7f, let-7g, let-7i, miR-100 and miR-210), upregulating cellular and EV miR-210. CSE-induced exosomal miR-210 promoted myofibroblast differentiation via silencing of ATG7 gene and upregulation of collagen type I and a-SMA expression in lung fibroblasts.
Liu et at., 2024 [68]	Mouse model (C57BL/6 J) and human cell line (BEAS-2B)	Human cardiomyocytes (AC16), and Murine cardiomyocyte	Serial Ultracentrifugation: 300× g 20min, 3,000× g 20min, 10,000× g 60min, 100,000× g 60-120min	NTA, Western Blot	Nicotine-induced LE-EVs were enriched in ERK5. Delivery of ERK5 to cardiomyocytes induced LDH release, GATA4, caspase 1 and 11

expression and the secretion of pro-fibrotic markers ILB1, IL6, TGFB, AKT, SMAD3. In cardiac tissue NIC-induced LE-EVs increased levels of collagen deposition; cleaved caspase-1 and 110, and pro-fibrotic markers.

Table S6. Scoring of papers studying the potential of CSE-induced LE-EVs to modulate recipient cell responses.

First Author and Year	Model (n/10)	Robustness of Model		Sample Size		Preparation of Exposure (n/3	EV isolation (n/3)	EV Charactерisation (n/4)	Bias Score
		Murine Model (n/2)	Cell Culture (n/4)	Murine Model (n/3)	Cell culture (n/2)				
Mekala et at., 2024 [59]	Human Cell culture (3)		Transformed cell line, fully defined exposure (2)		3 or more repeats per experiment (2)	ECVC preparation not clearly described (0)	Precipitation—EXOQuick-TC (3)	Multiple complementary techniques and suitable controls (3)	50.00%
Malyla et at., 2023 [60]	Human Cell culture (3)		Transformed cell line, fully defined exposure (2)		3 or more repeats per experiment (2)	CSE preparation is clearly described (1) and used within 1 hour of being prepared (1)	Serial UC (1)	Multiple complementary techniques, suitable controls and additional biomarkers (4)	53.85%
Bai et at., 2021 [61]	Human Cell culture (3)		Transformed cell line, fully defined exposure (2)		3 or more repeats per experiment (2)	CSE preparation is clearly described (1), absence of the filter is clearly stated (1) and used within 1 hour of being prepared (1)	Precipitation—EXOQuick-TC (3)	One method utilized (1)	53.85%
Song et at.,	Murine Cell		Transformed cell		Number of repeats	CSE preparation is clearly described (1),	exoEasy Maxi Kit (3)	Multiple complementary	50.00%

2021 [62]	culture (1)	line, fully defined exposure (2)	not specified (0)	absence of the filter is clearly stated (1) and used within 1 hour of being prepared (1)		techniques, suitable controls and additional biomarkers (4)	
Xu et at., 2018 [63]	Human Cell culture (3)	Transformed cell line, fully defined exposure (2)	3 or more repeats per experiment (2)	CSE preparation is clearly described (1), absence of the filter is clearly stated (1) and used within 1 hour of being prepared (1)	Precipitation—EXOQuick-TC (3)	Multiple complementary techniques, suitable controls and additional biomarkers (4)	65.38%
Dai et at., 2024 [64]	Human Cell culture (3)	Transformed cell line, fully defined exposure (2)	Number of repeats not specified (0)	CSE preparation is clearly described (1), absence of the filter is clearly stated (1) and used within 1 hour of being prepared (1)	Precipitation—EXOQuick-TC (3)	Multiple complementary techniques, suitable controls and additional biomarkers (4)	57.7%
Benedikter et at., 2019 [65]	Human Cell culture (3)	Transformed cell line, fully defined exposure (2)	3 or more repeats per experiment (2)	CSE preparation is clearly described (1), absence of the filter is clearly stated (1) and used within 1 hour of being prepared (1)	Ultrafiltration and Sepharose CL-4B SEC (3)	Multiple complementary techniques, suitable controls and additional biomarkers (4)	65.38%
Liu et at., 2016 [66]	Human Cell culture (3)	Transformed cell line, fully defined exposure (2)	3 or more repeats per experiment (2)	CSE preparation is clearly described (1)	Precipitation—EXOQuick-TC (3)	Multiple complementary techniques and suitable controls (3)	53.85%

Fujita et al., 2015 [67]	Human Cell culture (3)		Exposure of primary cells partially defined (3)		3 or more repeats per experiment (2)	CSE preparation not described (0)	Serial UC (1)	Multiple complementary techniques, suitable controls and additional biomarkers (4)	50 %
Liu et at., 2024 [68]	Human Cell culture (3), Animal model (2)	Exposure fully defined (2)	Transformed cell line partially defined exposure (1)	6 animals per group (2)	3 or more repeats per experiment (2)	PG/VG ratio clearly stated (1)	Serial UC (1)	Multiple complimentary techniques (2)	51.61%

Search Terms Strategy

Pubmed

((Epithelial OR Epithelium OR Epithelial cells) AND (Extracellular Vesicles OR EVs OR microvesicle OR exosome OR ectosome OR shedding vesicle OR microparticle) AND (lung) AND (Cigarette smoke OR smoking OR tobacco smoke OR tobacco OR e-cigarette OR e-cigarette vapour OR e-cig OR e-cigarette vapor OR vaping))) NOT review)

EMBASE

((Epithelial or Epithelium or Epithelial cells) and (Extracellular Vesicles or EVs or microvesicle* or exosome* or ectosome or shedding vesicle or microparticle) AND (lung) and (Cigarette smoke or smoking or tobacco smoke OR tobacco OR e-cigarette or e-cigarette vapour or e-cig or e-cigarette vapor or vaping))

Web of Science

(TS=(Epithelial OR Epithelium OR Epithelial cells) AND TS=(Extracellular Vesicles OR EVs OR microvesicle* OR exosome* OR ectosome OR Shedding Vesicle OR Microparticle) AND TS=(lung) AND TS=(Cigarette smoke OR smoking OR tobacco smoke OR tobacco OR e-cigarette OR e-cigarette vapour OR e-cig OR e-cigarette vapor OR vaping))
